# Comparative efficacy of acupuncture-related techniques for mild cognitive impairment: A Bayesian network analysis

**DOI:** 10.3389/fneur.2022.942682

**Published:** 2022-11-15

**Authors:** Xin Li, Lanfeng Lai, Liming Lu, Liang Yan, Kelin Deng, ZhiMing Li, Nenggui Xu, JiaYing Zhao

**Affiliations:** ^1^South China Research Center for Acupuncture and Moxibustion, Medical College of Acu-Moxi and Rehabilitation, Guangzhou University of Chinese Medicine, Guangzhou, China; ^2^Clinical Research and Big Data Laboratory, South China Research Center for Acupuncture and Moxibustion, Medical College of Acu-Moxi and Rehabilitation, Guangzhou University of Chinese Medicine, Guangzhou, China; ^3^Medical College of Acupuncture-Moxibustion and Rehabilitation, Guangzhou University of Chinese Medicine, Guangzhou, China

**Keywords:** acupuncture, network meta-analysis, mild cognitive impairment, acupuncture—therapy, systematic (literature) review

## Abstract

**Background:**

A comparison and ranking of the clinical effects of various acupuncture and acupuncture-related therapies on patients with mild cognitive impairment.

**Methods:**

Using network meta-analysis, we assessed the direct and indirect evidence from relevant research. Seven databases [PubMed, Web of Science, Cochrane Library, EMBASE, China National Knowledge Infrastructure (CNKI), VIP Database, and Wanfang database] were examined to find randomized controlled trials of acupuncture-related therapies for individuals with mild cognitive impairment. Two researchers independently reviewed the literature, retrieved the data, and evaluated the risk of bias in the included studies. The data were analyzed using Stata15.0 and R3.6.1 software.

**Results:**

A total of 27 randomized controlled trials involving 2,210 patients were included. Bayesian NMA showed that manual acupuncture combined with conventional therapy, moxibustion combined with conventional therapy, manual acupuncture, and electroacupuncture were most effective in improving the MMSE score. The most effective interventions related to the MoCA score were moxibustion combined with conventional therapy, followed by manual acupuncture combined with conventional therapy, acupressure combined with conventional therapy, and manual acupuncture combined with moxibustion. Manual acupuncture combined with moxibustion was dominant in the cluster ranking. The results of the node splitting method revealed that direct and indirect evidence were consistent (*P* > 0.05). In addition, publication bias was detected.

**Conclusion:**

This research will add to the body of knowledge about the safety and efficacy of acupuncture-related therapies in the treatment of mild cognitive impairment. The results of this study will also assist in the choice of clinical guidelines that optimize acupuncture treatment for patients with mild cognitive impairment.

## Introduction

The core cognitive abilities of people, such as memory, reaction time, visuospatial ability, and executive cognitive functions, decline as they age (1). Individuals with mild cognitive impairment (MCI), not exceeding the threshold for dementia diagnosis, are characterized as having focal or multifocal cognitive impairment with limited impact on activities of daily living (ADL) (2). According to a study in the United States (3), the 2020 US Census-adjusted prevalence of clinical Alzheimer's disease (AD) was 11.3% (95% confidence interval [CI] = 10.7–11.9). The study also demonstrated that in 2020, 6.07 million people (95% CI = 5.75–6.38) were living with clinical AD, and this is predicted to increase to 13.85 million (95% CI = 12.98–14.74) by 2060. MCI is a neurocognitive disorder with a higher prevalence than dementia and can be considered a risk factor for dementia (4, 5). While some individuals with mild cognitive impairment appear to stabilize or return to normal over time, more than half of those with moderate cognitive impairment develop dementia within 5 years (6, 7). Severe cognitive impairment occurs as MCI progresses to dementia, which substantially impacts peoples' daily life and livelihoods (8).

Given the lack of drug therapy options, at least 40% of MCI patients have increasingly turned to complementary and alternative therapies to alleviate their symptoms (9, 10). Research in recent years has focused on alternative medicines to improve outcomes for patients. Alternative treatments for MCI, such as acupuncture, tai chi, CHEIs, placebo, exercise, cognitive training, golf training, music therapy, and Vitamin E, have been shown to provide possible clinical or theoretical benefits for patients (11). Acupuncture-related therapy is one of the most often used adjuvant therapies for MCI patients (12), and according to a previous NMA study, acupuncture-related therapy may be the best and safest complementary and alternative therapy for improving cognitive function in people with Alzheimer's disease (13).

Acupuncture-related therapy has been widely used to treat MCI, with many studies confirming its efficacy (14–16). However, due to the great diversity of acupuncture-related therapies, few studies have directly compared different acupuncture techniques; it remains unclear which acupuncture method is optimal for treating MCI. As a result, determining the most effective acupuncture treatment for MCI is a challenging task. Although the term “acupuncture” has been given many different definitions, we have used the definition provided by the World Health Organization (17), which states that acupuncture means “needle pricking.” However, acupuncture therapy could also entail applying different forms of stimulation to specific areas. The term “acupuncture” will be used broadly throughout this essay to refer to any type of conventional acupunctures that replicate specific spots using needles, lasers, electricity, or pressure. Acupressure, ear (auricular) acupuncture, auricular pressure, scalp acupuncture, Bee Venom Acupuncture (BVA), conventional body needling, manual acupuncture, electroacupuncture, acupoint catgut embedding, and electroacupuncture are the specific acupuncture methods covered in this manuscript (18). The term “acupuncture-related therapies” refers to practices such as warm needling, acupoint injection, hydroacupuncture, or herbal decoction that combine moxibustion or medication with acupuncture (19).

To provide a comprehensive evaluation of the use of various acupuncture techniques in MCI patients, this study covers trials evaluating a number of different types of acupuncture procedures. NMA was utilized to assess and rank the best acupuncture treatment for MCI based on data from a variety of available databases.

## Methods

### Protocol and registration

The data gathering and analysis protocol used in this study was derived from the Preferred Reporting Items for Systematic Review and Meta-Analysis Protocols (20), and the protocol has been registered on the INPLASY website (registration number: INPLASY202240140: https://inplasy.com/inplasy-2022-4-0140/).

### Search strategy

The Bayesian Network Meta-Analyses statement and the Cochrane handbook were used to conduct this study (21). PubMed, Web of Science, EMBASE, China National Knowledge Infrastructure (CNKI), Cochrane Library, VIP Periodical Resource Integration Service Platform, and Wangfang Databases were searched from 4 March 2002 until 4 March 2022, with a combination of MeSH terms and free words.

### Study selection and intervention definitions

#### Types of studies

All randomized controlled trials (RCTs) of acupuncture-related therapies for MCI were used with either English or Chinese as the language option, regardless of blinding, publication, status, or length of the trial. Non-randomized uncontrolled trials were excluded. Additionally, case reports, animal experiments, individual cases, research advances, expert experience, conference articles, and duplicate articles were excluded.

#### Participants

The participants in this study were MCI patients who had been diagnosed with neurodegeneration rather than mild cognitive changes relating to potential causes, such as metabolic, vascular, and systemic. Patients with psychiatric disorders, such as those defined by the American Psychiatric Manual of Psychiatry and Statistics diagnostic criteria for MCI (22), the MCI Clinical Diagnostic Standards revised by Petersen in 2018 (23), the Reference Standard of Deficiency Syndrome Differentiation in traditional Chinese Medicine (24), and the 2018 Chinese Guidelines for the Diagnosis and Treatment of Dementia and Cognitive Impairment, were excluded (25).

#### Interventions

Studies that used a combination of acupuncture-related therapies and conventional therapy (CT) were included in the study. Classic acupuncture (with or without electrical stimulation and manual or scalp acupuncture), ear (auricular) acupuncture, auricular pressure, Bee Venom Acupuncture (BVA), abdominal acupuncture, acupoint catgut embedding, and acupressure, moxibustion (including direct and indirect moxibustion, and warm needling), and acupoint injection were all included in the definition of acupuncture-related therapies. The control group received conventional therapy or a placebo (sham acupuncture or other placebo treatments). These interventions were used alone or in combination. The current conventional-therapy strategy for MCI consists of conventional medicines (including Donepezil, Nimodipine, Huperzine, Perphenazine, Duxil, and Hydergine) and cognitive training.

#### Outcomes

We used the MoCA, MMSE, and ADL scales as the outcome indicators to assess the effects of acupuncture on cognitive function. The main outcomes were global cognitive function and behavioral abnormalities, which were assessed using recognized and standardized scales, such as the Mini-mental State Examination (MMSE) score, the Montreal Cognitive Assessment (MoCA) score, and the Activity of Daily Living (ADL) score.

### Data extraction and quality assessment

Two reviewers screened the full texts, extracted the correlated information from all the included studies, and cross-checked the data for consistency and accuracy of the extraction. We extracted the general information (e.g., publication year, author, sample size, gender, and age), intervention measures (e.g., experimental group: the types, acupoints, frequency and duration of acupuncture-related therapies; control group: conventional treatment, placebo or conventional treatment plus other acupuncture therapy), and outcomes (MMSE, MoCA, and ADL). The reviewers independently assessed the risk of bias in the studies using Cochrane's risk of bias tool 5.1.0 to evaluate the methodological quality of the research (26). The Cochrane Risk of Bias tool contains seven items: (1) random sequence generation; (2) allocation concealment; (3) blinding of participants and personnel; (4) blinding of outcome assessment; (5) incomplete outcome data; (6) selective reporting; (7) other sources of bias. Each trial was graded as either “low”, “high”, or “unclear” risk. A third reviewer resolved conflicts during trial selection when data extraction and quality evaluation scores were inconsistent.

### Statistical analysis

Given the potential for clinical heterogeneity among the included studies, the datasets were merged using a random effect model (21). The Cochrane risk of bias tool 5.1.0 was used to assess the quality of the studies. The Bayesian hierarchical model inference with Markov Chain Monte Carlo (MCMC) algorithm was employed in the Bayesian meta-analysis and was performed with R software (version 3.6.3) (27) and STATA (version 14.0) (28). The Aggregate Data Drug Information System (ADDIS Version1.16.8) package was used in the R software. This software uses the Bayesian framework as a base combined with the Markov chain Monte Carlo method to evaluate research data. We used 200,000 iterations, and the first 5,000 iterations served as a burn-in for annealing to eliminate the impact of the initial value. Four chains yielded 20,000 iterations and a factor of 2.5. The two models for estimating the ADDIS effect size were consistency and inconsistency. A consistency assessment calculated the ranking probability for all the interventions and made judgments about the sizes of the interventions that were included. By using node-splitting analysis and an inconsistent model, the consistency test was evaluated. When the node-splitting *p*-value was higher than 0.05, a consistency mode was chosen—if not, an inconsistency model was applied. Otherwise, continuous outcomes were represented by standardized mean differences (SMDs) and 95% confidence intervals (CIs). The model convergence was assessed using the potential scale reduction factor (PSRF). The convergence improved as the PSRF value approached 1. If the PSRF value was < 1.2, the model convergence was nevertheless considered satisfactory. We calculated the ranking probability for every intervention for every potential rank. Acupuncture methods were ranked by the surface under the cumulative ranking curve (SUCRA). Finally, a cluster ranking plot was created to assess the comprehensive ability of acupuncture methods to relieve symptoms in MCI patients.

## Results

### Characteristics of study

**Figure 1:**
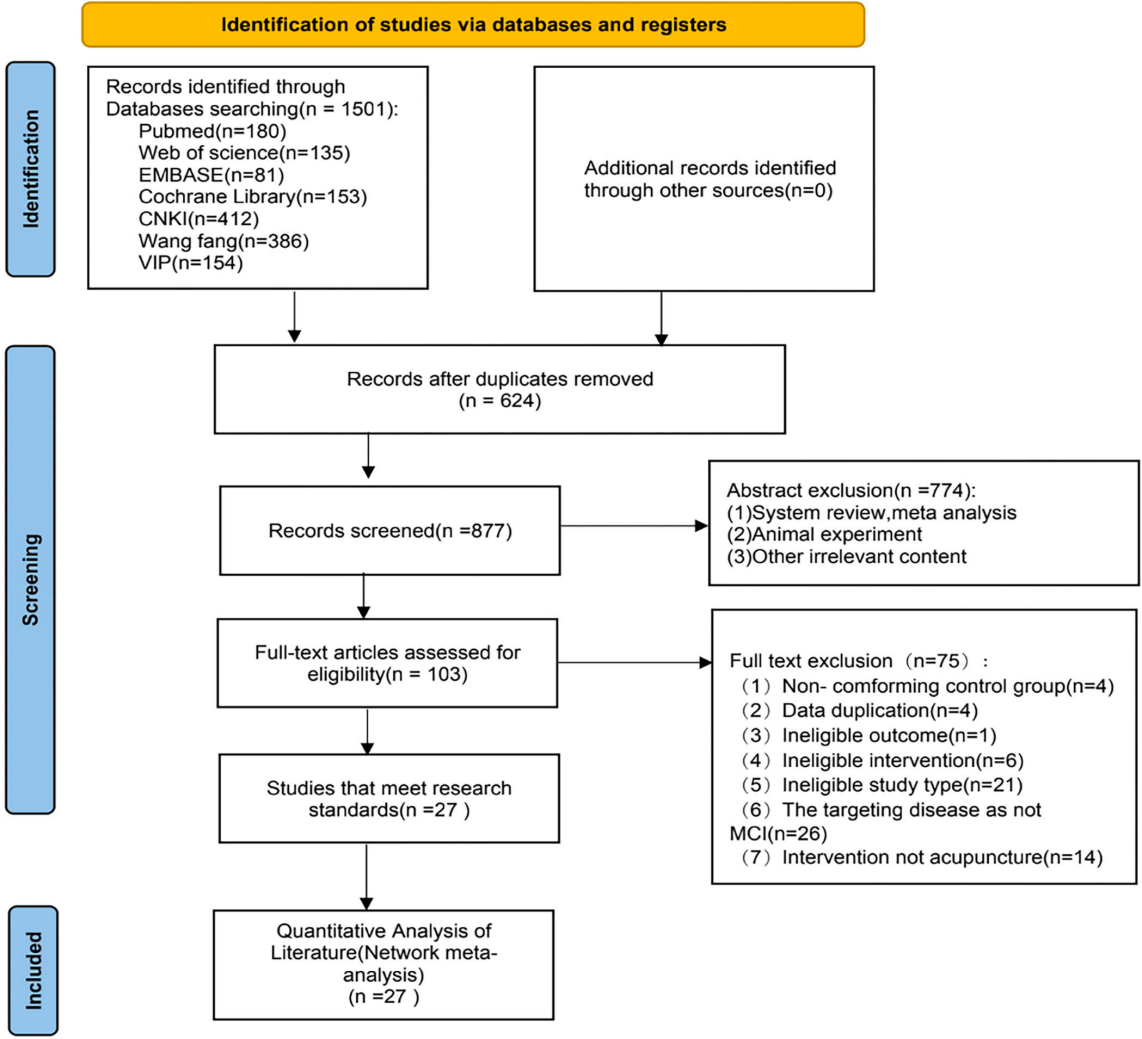
Preferred reporting items for systematic reviews and network meta-analyses (PRISMA) flowchart of the literature inclusion process. CNKI, China national knowledge infrastructure; VIP, Chinese scientific journals database; *n*, number of publications.

A total of 1,501 articles of probable relevance were obtained with 624 duplicates. The PRISMA flow diagram of the search process is shown in Figure 1. Seven hundred and seventy-four studies were excluded after a preliminary review of the titles and abstracts for the following reasons: unconnected studies (*n* = 495); animal experiments (*n* = 205); system reviews and meta-analysis (*n* = 74). A total of 103 RCTs were retrieved for full-text evaluation. The retrieved articles were analyzed using deep reading. A total of 75 records were excluded from the study based on the following criteria: (1) non-conforming control group (*n* = 4); (2) data duplication (*n* = 4); (3) the targeting disease was not MCI (*n* = 26); (4) ineligible study type (*n* = 21); (5) ineligible intervention (*n* = 6); (6) ineligible outcome (*n* = 4); (7) intervention was not acupuncture (*n* = 14). Finally, 27 studies (16, 29–54) were included in this study (Table 1).

**Table 1:** Main characteristics of included RCTs.

**Study**	**Country**	**Arms**	**Group**	**Sample size (n)**	**Average age (years)**	**Interventions**	**Acupoints**	**Frequency (t/w)**	**Retention (min)**	**Follow-up**	**Aderverse events**	**Measurement time points**	**Outcomes**
Chen et al.	China	2	G1	32	71 ± 5	SA	GV20,GV24,GV29,EX-HN1	3	30	√	×	8w	➀➁
			G2	32	71 ± 5	MA	GV20,GV24,GV29,EX-HN1	3	30			8w	➀➁
Yang	China	2	G1	40	71.61 ± 8.166	CT	/	7	NA	√	×	8w	➀
			G2	40	73.51 ± 8.964	MO + CT	GV20,CV8,K13	4 + 7	20			8w	➀
Chen et al.	China	2	G1	30	70.23 ± 6.92	CT	/	7	NA	×	×	4w	➀➁➂
			G2	30	67.77 ± 5.96	MA + CT	BL23,ST25,ST41,K13	6 + 7	40~60			4w	➀➁➂
Huang	China	2	G1	40	67 ± 3	CT	/	21	NA	×	×	12w	➀➂
			G2	40	68 ± 3	MA	GV20,GV24,GV29,GB13,EX-HN1,BL15	6	30			12w	➀➂
Yu	China	2	G1	56	70.170 ± 4.475	CT	/	21	NA	√	√	4w	➀➂
			G2	56	68.309 ± 7.333	EA	GV20,EX-HN1,GB20,GV24	3	30	√	√	4w	➀➂
Cao	China	2	G1	30	NA	CT	/	6	40	×	×	8w	➁
			G2	30	NA	CT + MA	/	6	360			8w	➁
Wang et al.	China	2	G1	30	68.43 ± 6.16	CT	/	6	30	×	×	8w	➁➂
			G2	30	66.83 ± 6.72	CT + MA	/	6	30			8w	➁➂
Wang	China	2	G1	30	54.43 ± 3.081	CT	/	18	NA	×	√	1w	➀➁➂
			G2	30	53.43 ± 2.725	CT + MA	GV26,GV24,GV20	6 + 2	30			1w	➀➁➂
He et al.	China	2	G1	30	66 ± 6	CT	/	7	NA	×	×	12w	➀➁➂
			G2	30	64 ± 7	WA	GV20,GV14,GV9,GV4	7	60			12w	➀➁➂
Zhu et al.	China	2	G1	30	NA	CT	/	18	NA	×	×	5w	➀➁➂
			G2	30	NA	MA + MO	CV4,GV4	6	30			5w	➀➁➂
Wang et al.	China	2	G1	105	70.3 ± 9.4	CT	/	7	NA	√	√	8w	➀➁
			G2	105	72.3 ± 8.6	MO	GV20,CV8,K11	4	20			8w	➀➁
Liu	China	2	G1	18	69.32 ± 6.86	CT	/	7	NA	×	×	30d	➀➁
			G2	18	66.00 ± 6.84	EA	GV20,GB20,BL23,GB39,K13	7	NA			30d	➀➁
Liu et al.	China	2	G1	9	77 ± 6	CT	/	7	NA	×	×	4w	➀
			G2	8	73 ± 8	EA	EX-HN1,GB20,BL23,HT7,GB39,K13	7	20			4w	➀
Jin	China	2	G1	16	73.67 ± 3.266	CT	/	21	NA	×	×	45d	➁
			G2	14	72.54 ± 7.067	EA	EX-HN1,BL23,GB20,K13	7	20			45d	➁
Li et al.	China	2	G1	39	61.9 ± 6.8	CT	/	7	NA	×	×	3m	➀➁
			G2	39	62.8 ± 5.9	CT + EA	GV16,GV23,BL62,PC7	3.5	30 min			3m	➀➁
Li	China	3	G1	32	61.14 ± 9.05	CT	/	7	NA	×	√	8w	➀➁
			G2	32	65.10 ± 8.76	MO	GV20,CV8,K11	3.5	20 min			8w	➀➁
			G3	32	64.87 ± 9.23	MO + CT	GV20,CV8,K11	3.5	20 min			8w	➀➁
Su	China	2	G1	35	67.05 ± 6.10	CT	/	3.5	NA	×	√	4w	➀➁
			G2	35	63.85 ± 6.32	MO	GV20,CV4,ST36,GB39	7	NA			4w	➀➁
Liu	China	2	G1	65	70.24 ± 9.378	CT	/	7	NA	×	√	8w	➀➁
			G2	65	72.15 ± 8.418	MO	GV20,CV8,K11	3.5	20 min			8w	➀➁
Yu et al.	China	2	G1	32	66.75 ± 6.41	CT	/	7	NA	×	×	4w	➀➁➂
			G2	32	64.13 ± 6.02	MO	GV20,CV4,ST36,GB39	3.5	30 min			4w	➀➁➂
Du	China	2	G1	20	68.25 ± 5.80	CT	/	7	NA	×	×	8w	➀➁➂
			G2	20	69.03 ± 5.47	MA	GV20,GV14,GV23,GV24,GV16,GV15,EX-HN1,K13,ST36,GB39,K14	6	40 min			8w	➀➁➂
Yu	China	2	G1	25	63.32 ± 8.61	SA + CT		2	60 min	×	√	8w	➀➁➂
			G2	25	65.48 ± 7.47	MA + CT	GV20,EX-HN1,GV24,GV16,GV14,BL23,BL18	2	60 min			8w	➀➁➂
Zhu et al.	China	2	G1	30	74 ± 7	CT	/	21	NA	×	×	12w	➀➁
			G2	30	69 ± 7	MO	GV20,GV14,GV24,GV11	6	20 min			12w	➀➁
Zhao et al.	China	2	G1	30	58.14 ± 9.15	CT	/	21	NA	×	×	8w	➀➁
			G2	30	55.34 ± 8.48	MA + CT	GV20,EX-HN1,GV24,GB20,GB13	4	NA			8w	➀➁
Sun et al.	China	3	G1	45	NA	AP + CT	GV20,GB20,GV24,EX-HN1,EX-HN5	5 + 15	1 min 10 s	√	×	6m	➀➁
			G2	45	NA	CT	/	5	NA			6m	➀➁
			G3	45	NA	AP	GV20,GB20,GV24,EX-HN1,EX-HN5	15	1 min 10 s			6m	➀➁
kim jh,cho et al.	Korea	2	G1	16	67.25 ± 5.15	MA	GV20,EX-HN1,GB20,GV24	3	30	×	×	8w	➁
			G2	16	64.63 ± 4.47	EA	GV20,GV24,EX-HN1,GB20	3	30			8w	➁
Zhao et al.	China	2	G1	96	67 ± 6	EA	GV24,GV20,EX-HN1,GB20	3	30	√	√	4w	➀➂
			G2	96	69 ± 7	CT	/	21	NA			4w	➀➂
Wang et al.	China	2	G1	16	64.56 ± 5.25	SA	EX-HN1,GV29,PC6,K13,ST40,LR3	5	NA	√	×	4w	➀➁
			G2	16	65.88 ± 4.66	MA	EX-HN1,GV29,PC6,K13,ST40,LR3	5	NA			4w	➁

The studies included in this investigation comprised 27 RCTs, with 26 trials from China and one from South Korea. Twenty-two trials were published in Chinese (81.48%), and five in English (18.52%). There were 25 double-arm RCTs and two three-arm RCTs in the trials. The network analysis comprised 1,797 people from 25 two-arm studies and 231 from two three-arm studies. Seven of the 25 trials compared electro-acupuncture (EA) and conventional therapy (CT), five trials compared manual acupuncture (MA) and conventional therapy (CT), and the two-arm investigations focused on moxibustion (MO) vs. conventional therapy (CT). Three trials compared manual acupuncture (MA) combined with conventional therapy (CT), two trials compared manual acupuncture (MA) vs. Sham acupuncture (SA), and one trial compared manual acupuncture (MA) vs. electro-acupuncture (EA), warm acupuncture (WA) vs. CT, moxibustion (MO) combined with conventional therapy (CT) vs. conventional therapy (CT), and manual acupuncture (MA) combined with moxibustion (MO) vs. conventional therapy (CT). Acupressure (AP) combined with conventional therapy (AP+CT), moxibustion (MO) combined with manual acupuncture (MO + MA), and moxibustion combined with conventional therapy (MO + CT) were compared in two three-arm experiments. The MMSE was reported in 22 trials and the MoCA in 21 trials as an end measure. In addition to conventional drugs, cognitive training, and placebo, the research included 12 acupuncture intervention modalities. A summary of the detailed information from the 27 RCTs can be found in Table 1.

### Methodological quality assessment

**Figure 2:**
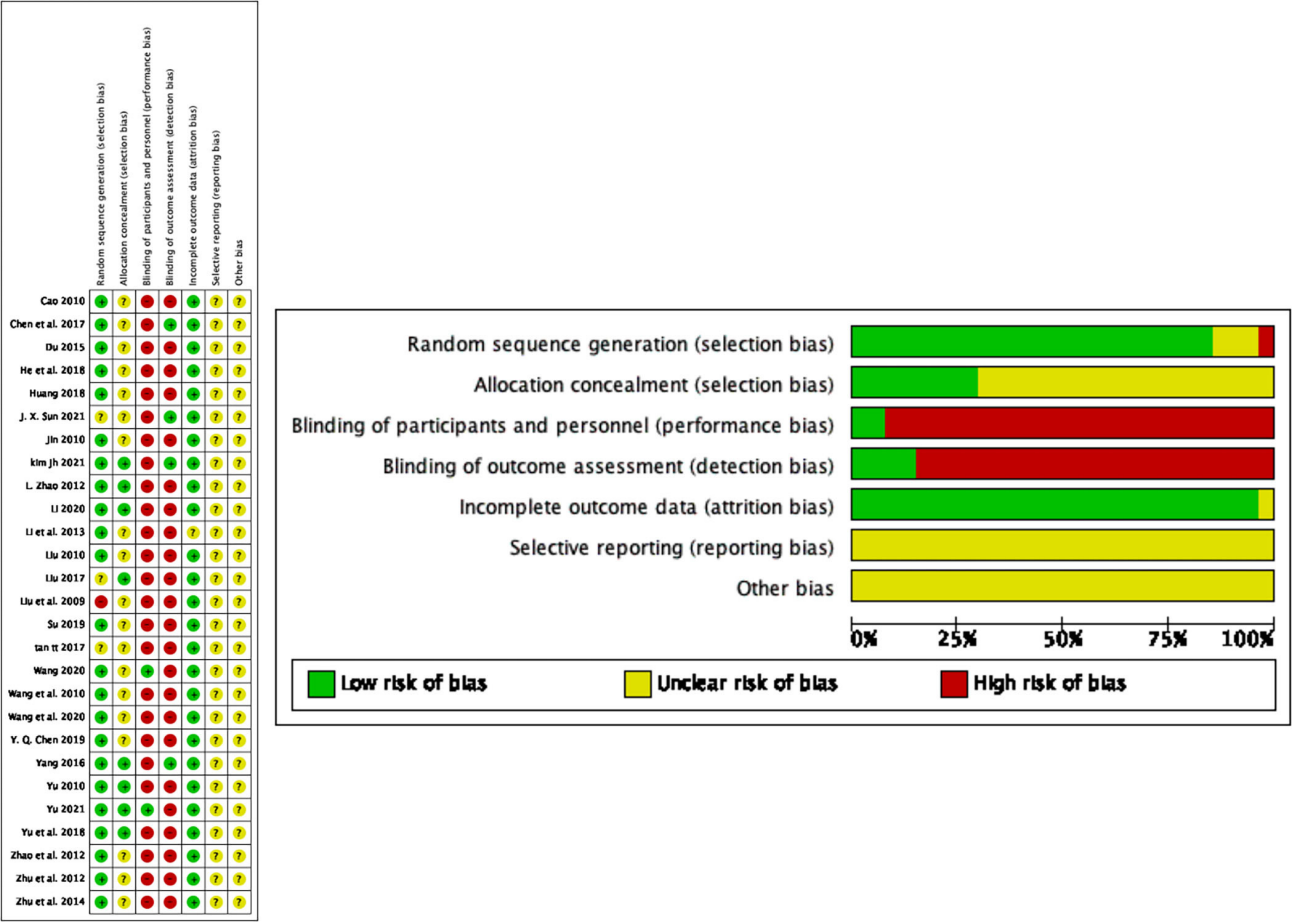
Risk of bias graph and summary.

Twenty studies randomized participants using a random number table, five studies used computer-generated random numbers, and two studies provided no information regarding the randomization procedure. Eight studies used sealed opaque envelopes for allocation concealment, whereas the remaining nineteen studies made no mention of their randomization procedures. Patients and researchers were only blinded in two trials. The result evaluators in four trials were blinded. Since the study did not explain why two examples were eliminated, one experiment was rated an “uncertain” risk of bias. The risk of bias for all studies with no indication of the study procedure or the influence of selective reporting was rated as “uncertain.” No other bias was detected in the included studies (Figure 2).

### Outcomes

#### Mini-mental state examination (MMSE) score

**Figure 3:**
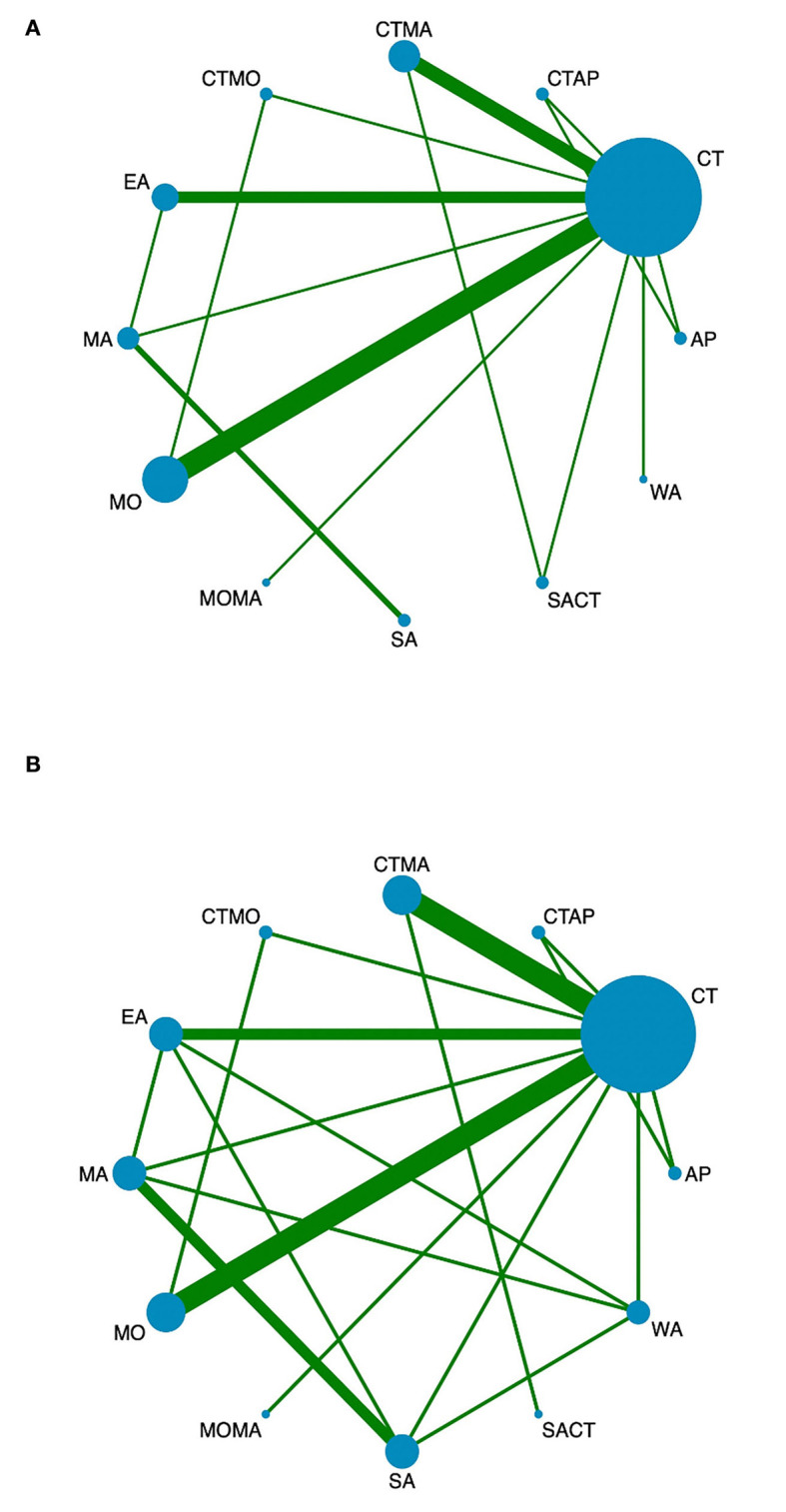
Network diagrams of comparisons of different outcomes of treatments in patients with MCI. **(A)** Network meta-analysis of multiple acupuncture-related treatments for MMSE. **(B)** Network meta-analysis of multiple acupuncture-related treatments for MoCA. Each node represents an intervention and the size of each node represents the number of randomly assigned participants. Each line represents a direct comparison between interventions and the width of the lines represents the number of studies. MMSE, mini-mental state examination; MoCA, montreal cognitive assessment.

**Figure 4:**
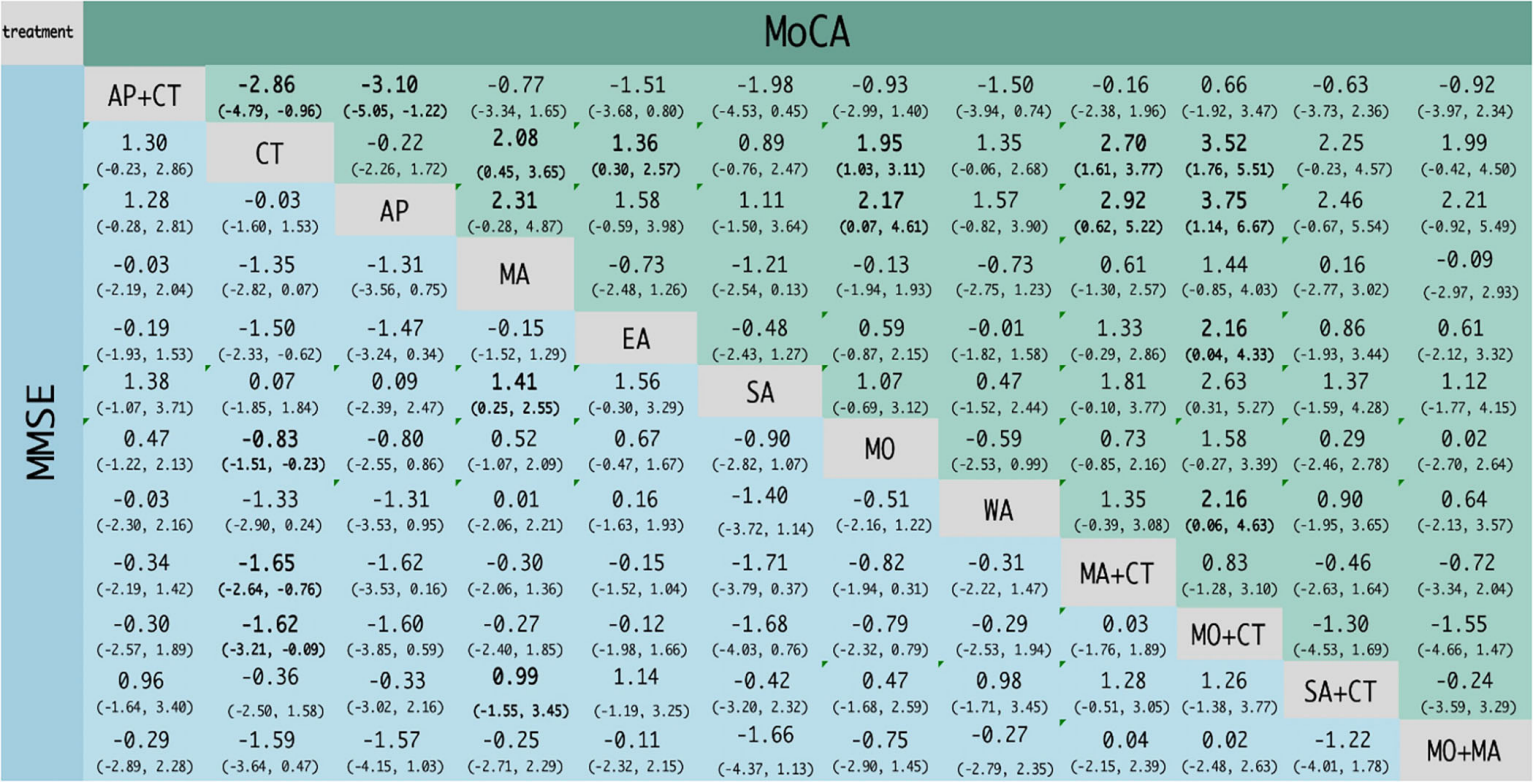
Network meta-analysis results for MMSE and MoCA scores. The bold font indicates a statistical difference.

**Figure 5:**
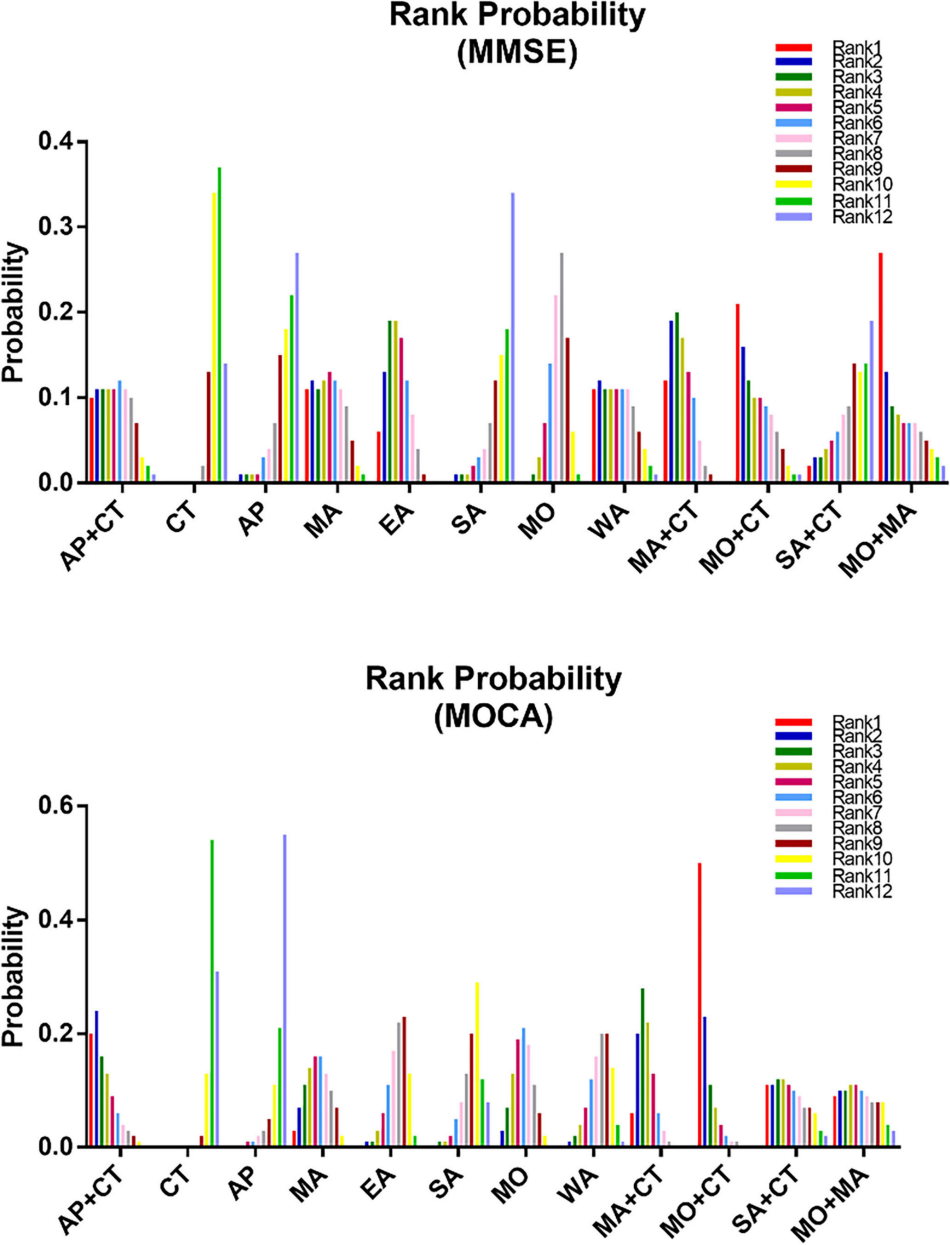
Rank probability plot of network meta-analysis for MMSE and MoCA.

**Figure 6:**
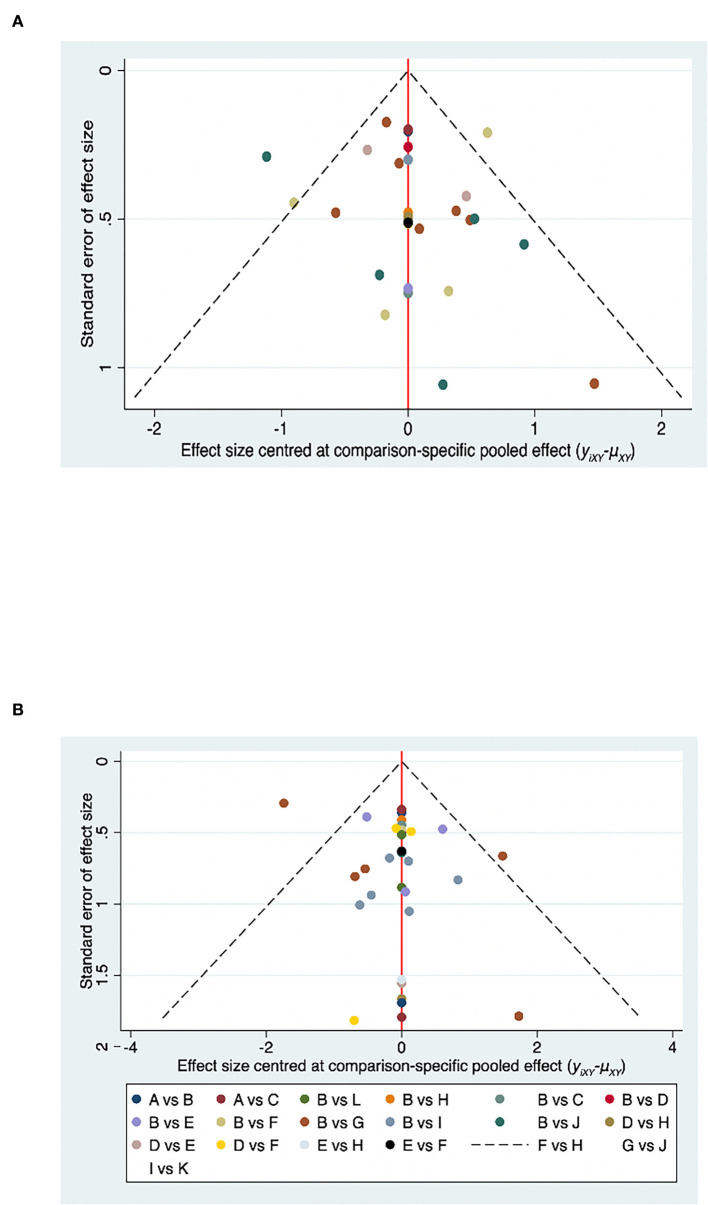
Funnel plot of MMSE and MoCA for the network meta-analysis. **(A)** Funnel plot of MMSE for the network meta-analysis. **(B)** Funnel plot of MoCA for the network meta-analysis.

**Figure 7:**
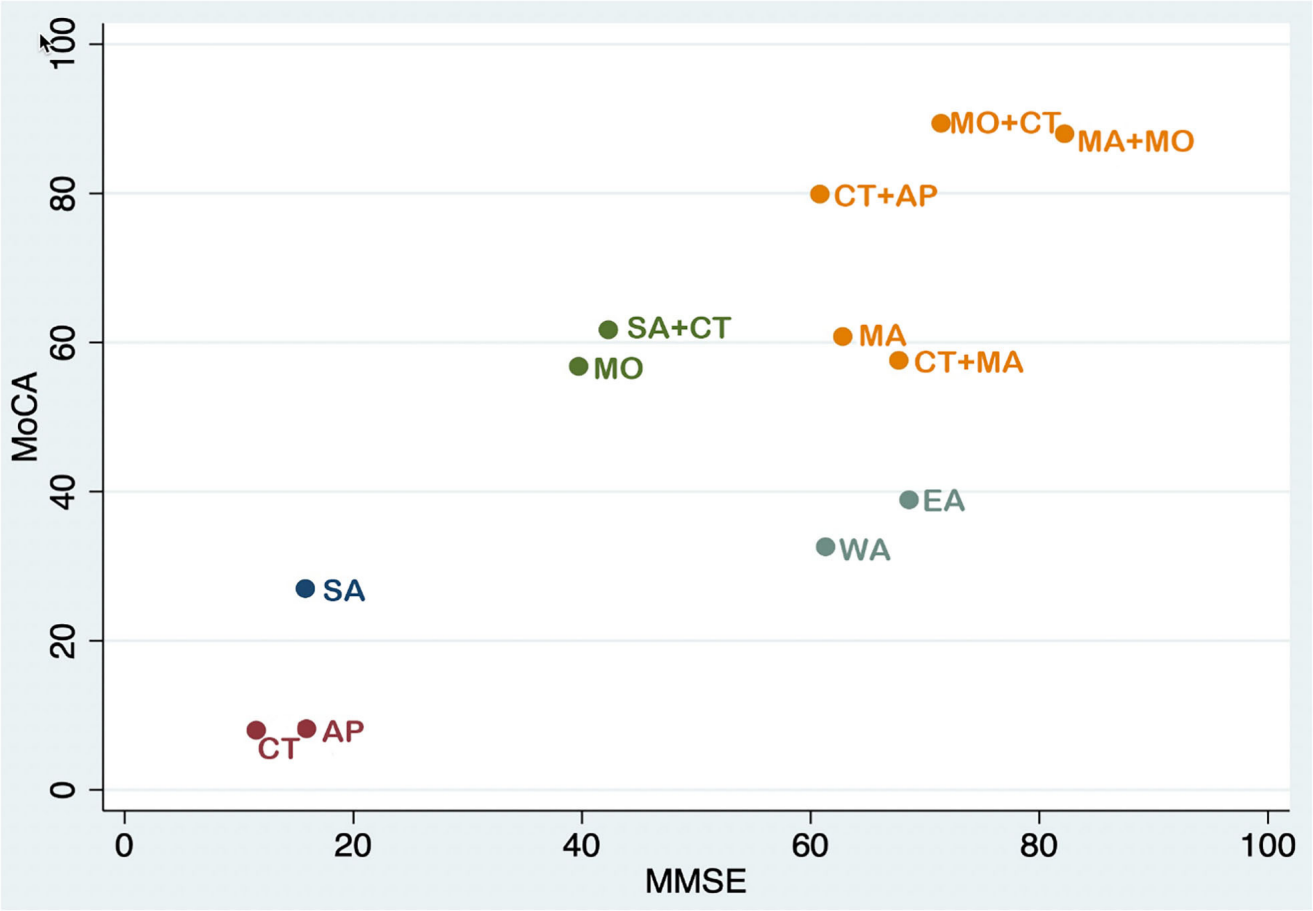
Cluster ranking plot in MMSE and MoCA scores. Each color represents a group of interventions that belong to the same cluster. Interventions lying in the upper right corner were more effective than the other interventions.

Twenty-two studies reported MMSE scores, resulting in four closed loops: AP-AP + CT – CT, MA – EA – CT, MO – MA + CT – CT, and MO – MO + CT – CT (Figure 3). Since the PSRF values tended to 1 and the incongruity model results were similar to the congruity model, which suggests the indicator stability and consistency were good, the MCMC congruity model was selected for network meta-analysis MMSE scores. Node-split was utilized to identify inconsistency and heterogeneity for the MMSE scores. Due to a *P*-value of >0.05, the direct evidence approximated the indirect evidence. The node-split plots demonstrate that each comparison's direct and indirect evidence had hardly any heterogeneity or inconsistency (Supplementary Figure S1). In the comparisons, SA was the least efficacious among all interventions (Figures 4, 5). The ranking probability of MMSE (Figure 6A) showed that MO + MA had the highest probability (28%) of being the best treatment for MCI, followed by MA + CT (22%) and MO + CT (14%). According to cumulative probability, MO + MA had the highest probability (82.2%) to be the most efficacious treatment for MCI (Figure 7).

#### Montreal cognitive assessment (MoCA)

Twenty-one studies reported MoCA scores, forming seven closed loops: AP–AP + CT – CT, MA – EA – CT, MA – EA – SA, MA – EA – WA, CT – SA – WA, CT – MO – MO + CT, and CT – MA + CT – SA + CT (Figure 3). The MCMC congruity model was used for network meta-analysis of MoCA scores since convergence assessment demonstrated that PSRF values tended to 1. The results of the incongruity model were identical to those of the congruity model, which suggests the indicators' stability and consistency were good. The node-split plots demonstrated that each comparison's direct and indirect evidence had hardly any heterogeneity or inconsistency in MoCA scores (Supplementary Figure S2). In the comparisons, CT was the least efficacious among all interventions (Figures 4, 5). The ranking probability of MoCA (Figure 6B) showed that MO + CT had the highest probability (52%) of being the best treatment for MCI, followed by MA + CT (28%) and MO + MA (9%). According to cumulative probability, MO + CT had the highest probability (91.4%) to be the best treatment for MCI (Figure 7).

#### Activity of daily living (ADL)

Activity of daily living (ADL) scores were used to assess self-care ability after the intervention. Four studies involving 124 participants in the treatment group and 124 in the control group assessed the ADL scale score (MD = 3.08, 95% CI [0.66, 5.49], *P* > 0.05). The data heterogeneity test (*I*^2^ = 94.8%, *I*^2^ > 70%) could not be used for the meta-analyses because of the high heterogeneity (Supplementary Figure S3). We also implemented sensitivity analysis by omitting a single study by step, and the result showed that one trial might be the likely source of heterogeneity, which changed the sensitivity analysis result when it was excluded from the pooled process (55, 56) (Supplementary Figure S4). After the study was removed, the robustness of the synthesized result changed (MD = 1.23, 95% CI [0.75, 1.70], *P* > 0.05) (Supplementary Figure S5), which showed that our result had high sensitivity and the treatment group combined with acupuncture-related therapy did not improve the ADL scale score.

### Publication bias

A comparison-adjusted funnel plot was used to analyze MMSE and MoCA publication bias. No publishing bias is present when the distribution points in the funnel plot are symmetric (Figure 7). Most of the points were evenly distributed on both sides of the midline and concentrated in the central area. The majority of the research included had moderate sample sizes, and the funnel plot indicated that these studies were biased to a low degree. However, a few locations outside the two dashed lines indicate that this research might be potentially heterogeneous.

### Adverse events

According to the studies utilized, adverse events were minor, did not require medical evaluation or specific intervention, and primarily consisted of skin irritation, slight headache, stomachache, and nausea from the application of the treatment. Out of 27 trials, adverse events were reported in nine studies. Adverse events were reported in five out of nine trials and were related to patients complaining of transient dizziness or headache after the first treatment. Two trials reported that a total of six subjects had slight skin reddening and mild swelling at the end of the stimulation procedure, and the side effects subsided spontaneously. Mild scalding of the skin also was observed in two trials after the first moxibustion treatment.

### Acupoint selection

**Figure 8:**
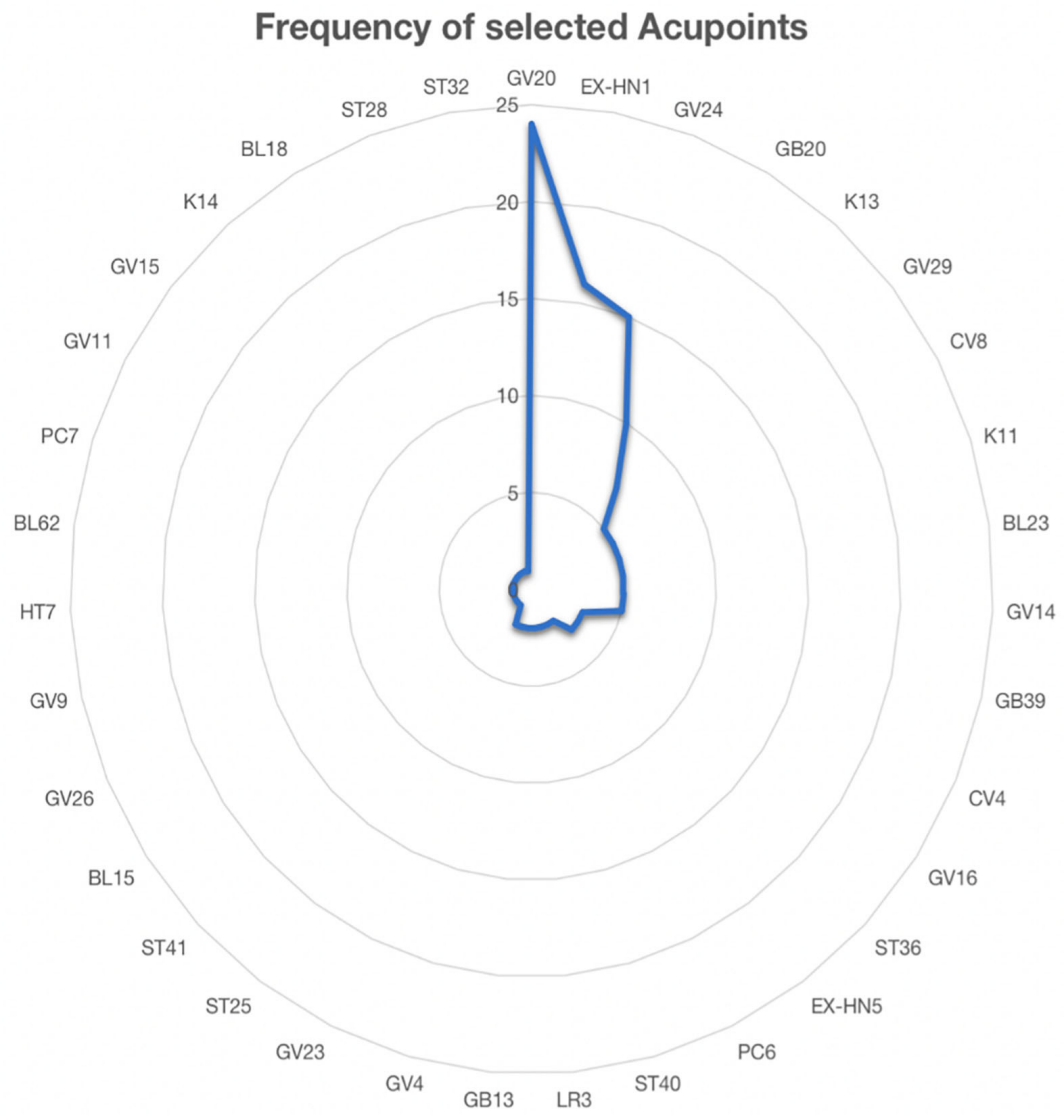
The frequency of selected acupoints for treatment.

Therapy patterns and classic acupuncture points were among the details we examined (Figure 8). The most frequently used acupoints were GV20 (85.71%), EX-HN1 (57.14%), GV24 (53.57%), and GB20 (35.71%), as shown in Figure 3. Even though researchers used various acupoint selection and combination techniques, most high-frequency acupoints for MCI treatment were focused on the craniofacial area. The original studies were all administered by hospital employees, with acupuncture treatments administered by medical professionals. The time of therapy varied between 30 and 360 min, with 30 min being the most common (*n* = 13). The shortest treatment trial lasted 7 days, while the longest lasted 3 months. Despite the fact that treatment intervals ranged from 7 to 90 days, more than 96% of studies (*n* = 26) favored a long therapeutic course of at least 4 weeks.

## Discussion

The study presented herein is, to the best of our knowledge, the first Bayesian NMA of acupuncture-related approaches for the treatment of MCI. Acupuncture has been demonstrated to be effective in the treatment of MCI, but there are also many different types of acupuncture. Low-curative-effect acupuncture methods worsen the condition of MCI patients and waste medical resources. As a result, we set out to find the best acupuncture therapy options for MCI in this Bayesian network to compare the effects of different acupuncture-related therapies. The study included 27 RCTs with a total of 2,210 subjects. MMSE in NMA indicated that of the 12 interventions evaluated, EA, MA combined with CT, MO combined with CT, and MO combined with MA had considerably more significant treatment effects than the other treatments. According to the results of MoCA in NMA, AP combined with CT, MA combined with CT, MO combined with CT, and MA also led to significantly increased beneficial effects. The node-splitting method revealed that the direct and indirect evidence supporting treatment efficacy was consistent. We were able to rate a large number of treatments by calculating similar probabilities and then ranking them from highest to lowest based on the method we employed. MO combined with MA ranked high in the MMSE, and MO combined with CT was shown to be the most effective treatment in MoCA. It is therefore noteworthy that the cumulative probability of MA combined with MO did not show a significant advantage over MO combined with CT in terms of MoCA. Due to individual variation, MoCA may not be the most suitable method for evaluating the clinical efficacy of TCM.

According to cumulative probability, the cluster ranking plot showed five categories of interventions (Figure 7). The following are a few key findings from the outcomes of the projects included in this study. The cluster ranking plot showed five categories of interventions that were based on cumulative probability. On the plot, the categories of SA combined with CT and MO are located in the left-middle, while the categories of EA and WA are situated near the right-middle. However, the impacts of these four acupuncture methods on MMSE and MoCA scores were rather limited, and these methods may not contribute to satisfactory clinical benefits. Favorable MMSE and MoCA scoring results are in the last group, which is placed in the upper right corner: MA, MA combined with CT, CT combined with AP, MO combined with CT, and MA combined with MO. The most significant of the outcomes was that MO combined with MA may have superior therapeutic efficacy in the treatment of MCI. MMSE and MoCA scoring results are preferred in the final group, which is placed in the upper right corner and includes the following therapies: MA, MA combined with CT alone, CT combined with AP, MO, MO combined with CT, and MA combined with MO. Based on our findings, we propose that clinical acupuncturists choose one of these three approaches as the primary therapy option for MCI patients. MA combined with MO appears to be the most effective treatment for improving MMSE and MoCA scores in MCI patients.

In TCM, acupuncture is a crucial component for controlling health. Its therapeutic benefits may be induced by stimulating certain meridian acupoints. Numerous investigations have shown that acupuncture can help with brain tissue healing in both human tests and animal studies, with reliable and repeatable results. Acupuncture is increasingly being utilized in clinical studies to treat dementia and cognitive impairments brought on by MCI disease, especially because of its safety when compared to pharmaceutical treatments. Acupuncture stimulation has reportedly been shown to potentially stimulate nerve fibers, enhance local blood flow, and stabilize and speed up cerebral metabolic responses in a variety of brain systems. Acupuncture may also improve LTP deficiencies and boost cerebral blood flow to lessen dementia symptoms, according to animal studies (57). A previous systematic review found that MA improved MoCA and MMSE scores in elderly patients with MCI, indicating that MA may have some therapeutic benefits for senior patients with MCI (58). Additionally, a clinical investigation using MA to treat MCI demonstrated that manual acupuncture was beneficial in enhancing memory and cognitive function in MCI patients (59). Moxibustion is an external treatment method used in traditional Chinese medicine. It has the effect of warming the meridians, dispersing cold, tonifying the deficient and consolidating the root, motivating Qi and blood, eliminating swelling and dispersing knots, and preventing diseases (60). Due to the deficiency of Qi in elderly MCI patients, Moxibustion has the effect of removing an obstruction from the meridians and collaterals. When the herb Artemisia vulgaris is burned over an acupoint, it produces a warm stimulation effect and is often regarded as an acupuncture treatment. It has proven to be fruitful when used in moxibustion intervention in MCI (61). Furthermore, MA and MO are frequently employed in research and therapeutic practice. Consequently, we recommend that clinical acupuncturists consider utilizing one of these approaches as the first therapy choice for MCI patients. Overall, MA combined with MO appears to be the most effective treatment for improving MMSE and MoCA scores in patients with MCI. Therefore, our study may provide an important clinical reference value for clinical investigations of acupuncture in the treatment of MCI and provide essential information to decision-makers.

The selection of acupuncture points is one of the essential factors for ensuring that acupuncture positively affects the patient. Despite the wide range of precise procedures in the included RCTs, a descriptive analysis of the data from the available studies showed the following acupuncture treatments to be effective for MCI. In our research, the most often used local points were Baihui (GV20), Si-shen-cong (EX-HN1), Shen-ting (GV24), and Feng-chi (GB20). The specific nature of acupuncture treatment makes it necessary to utilize studies that explore particular acupuncture manipulations. GV20 is a crucial point on the GV meridian, which receives all of the yang-qi transmitted by all of the body's meridians. It has been reported that GV20, EX-HN1, and GV24 are linked to the brain and play critical roles in influencing cerebral function, including cognition (62). It is believed in TCM that “the brain is the host of the mind,” which explains why the primary acupoints in our study were mainly chosen in the craniofacial region, whereas the auxiliary acupoints that can lift the spirit, clear the mind, or promote resuscitation were selected to be dispersed throughout the body (63). This pattern of acupoint selection and the combination is highly consistent with the theory of Traditional Chinese Medicine. Given that MCI is a chronic neurodegenerative disease, most researchers recommended a longer retention time, more frequent sessions, and a longer therapy course to guarantee appropriate acupuncture stimulation. A needle retention time of 30 min is also advised.

In addition, we reviewed nine trials that had documented adverse events. The most often reported adverse effects were increased minor headaches, skin irritation, and other symptoms. According to the findings, the needle fainting effect was the primary source of a slight headache, whereas conventional medications induced stomachache and nausea. Furthermore, the rate of adverse events was relatively high for both moxibustion and warm acupuncture. However, because of the limited sample sizes in the studies of these two intervention modalities, it is difficult to draw definitive conclusions about adverse events.

## Limitations

There were several limitations in this study. First and foremost, there was insufficient information on the effectiveness of acupuncture-related treatment for MCI due to the small sample sizes, the small number of patients in each trial, and the limited assessment of MMSE and MoCA data currently accessible. Second, a small number of randomized controlled trials (RCTs) revealed potential bias due to the small number of participants and the use of voluntary reporting. Fortunately, no apparent inconsistency or heterogeneity was found in this network meta-analysis. However, it is possible that some of the included articles overstated the efficiency of therapies, which could have had an impact on our findings if they had been excluded. Third, we have excluded several complementary and alternative therapy interventions due to our selection criteria for outcome measures, which may have impacted the quality of the evidence in this area. Furthermore, as for the use of Nimodipine and Donepezil in conventional therapy for MCI, some evidence from Chinese clinical articles shows that Donepezil and Nimodipine are used in the clinical treatment of MCI in some areas of China, but are rarely used in the USA and other parts of the world (52). Last, although the included studies were subjected to a full assessment using the Cochrane Collaboration's risk of bias methodology, the overall quality of the trials was not very good. This may be a result of using solitary blindness as a high-risk condition. Due to the particular nature of acupuncture research, single-blind designs are frequently used, which may have elevated the possibility of bias in the study findings.

## Conclusion

This study has demonstrated that manual acupuncture combined with moxibustion is the most effective treatment for improving MMSE and MoCA scores in patients with mild cognitive impairment. However, more robust comparative evidence is needed to confirm this conclusion. We suggest that more high-quality, large-sample, multicenter randomized controlled trials (RCTs) be done to confirm these findings.

## Data availability statement

The original contributions presented in the study are included in the article/Supplementary material, further inquiries can be directed to the corresponding authors.

## Author contributions

JZ and NX conceived and designed the study. XL and KD were involved in writing and draft preparation. LY and ZL were involved in literature inclusion and exclusion. XL was involved in writing, draft preparation, and supervision. All authors contributed to the manuscript and approved the submitted version.

## Funding

This work was supported by grants from General Program of National Natural Science Foundation Youth Program of China, China (grant number 81704168 to JZ), Natural Science Foundation of Guangdong Province, China (grant number 2017A030310359 to JZ), and Discipline Collaborative Innovation Team Program of Double First-Class and High-Level Universities for Guangzhou University of Chinese Medicine (grant number 2021XK01 to NX).

## Conflict of interest

The authors declare that the research was conducted in the absence of any commercial or financial relationships that could be construed as a potential conflict of interest.

## Publisher's note

All claims expressed in this article are solely those of the authors and do not necessarily represent those of their affiliated organizations, or those of the publisher, the editors and the reviewers. Any product that may be evaluated in this article, or claim that may be made by its manufacturer, is not guaranteed or endorsed by the publisher.
