# Supplementary figures and images for: Comparative efficacy of acupuncture-related techniques for mild cognitive impairment: A Bayesian network analysis
Source: Front Neurol. 2022 Nov 15;13:942682. doi: 10.3389/fneur.2022.942682 (PMC9706122; doi:10.3389/fneur.2022.942682)

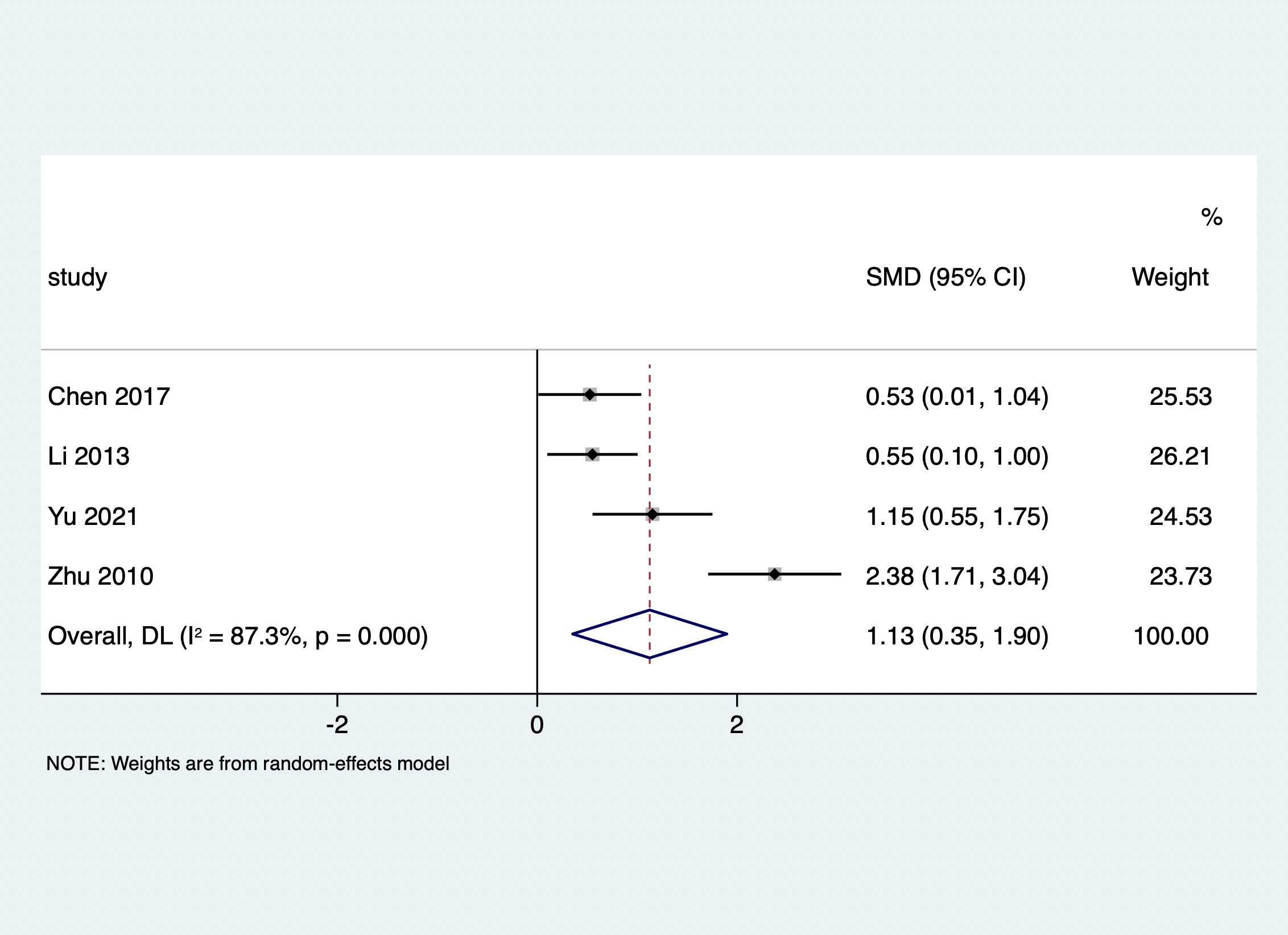

Supplement: Supplementary file 3 [file Image_3.jpeg]

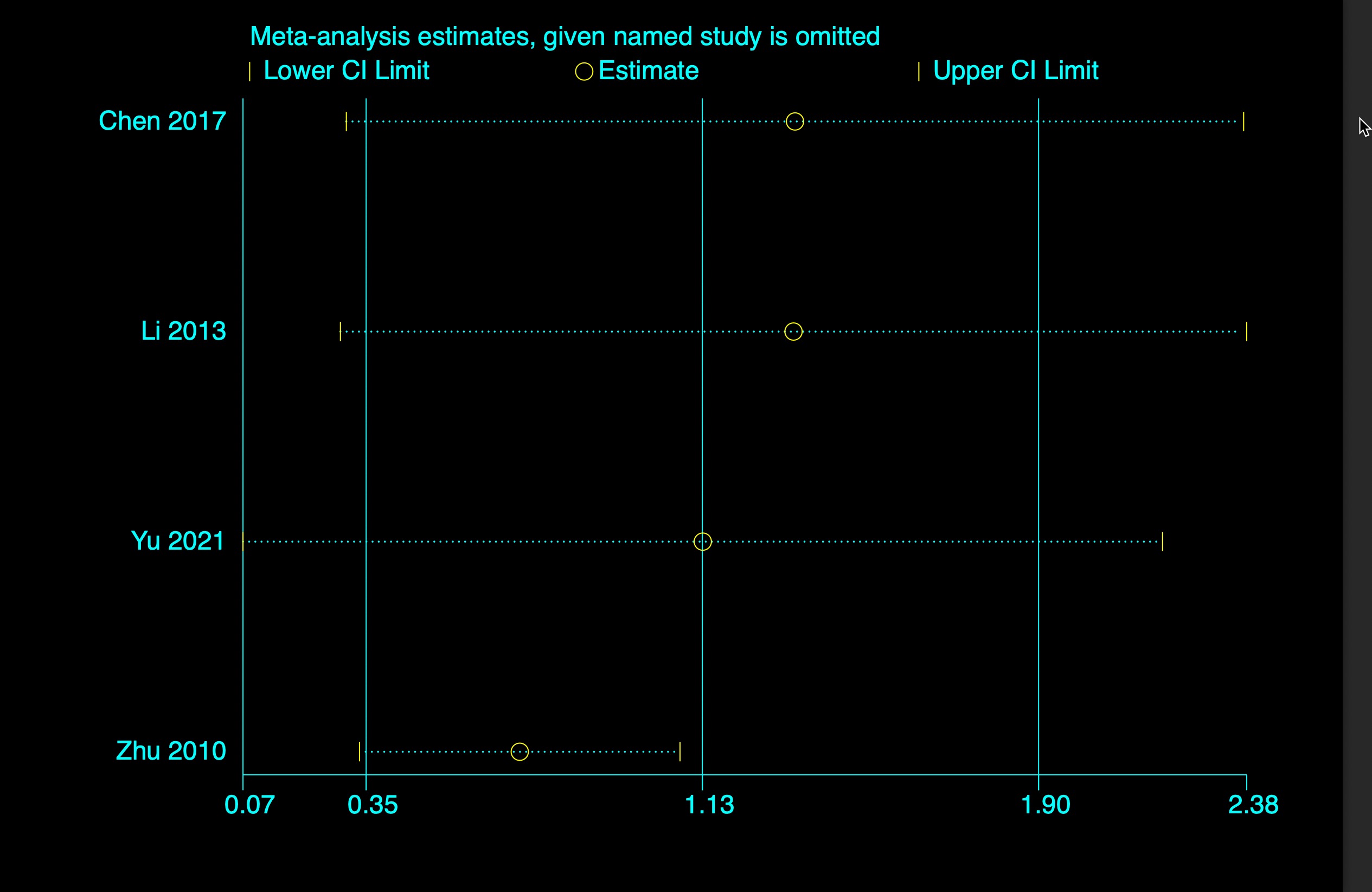

Supplement: Supplementary file 4 [file Image_4.jpeg]

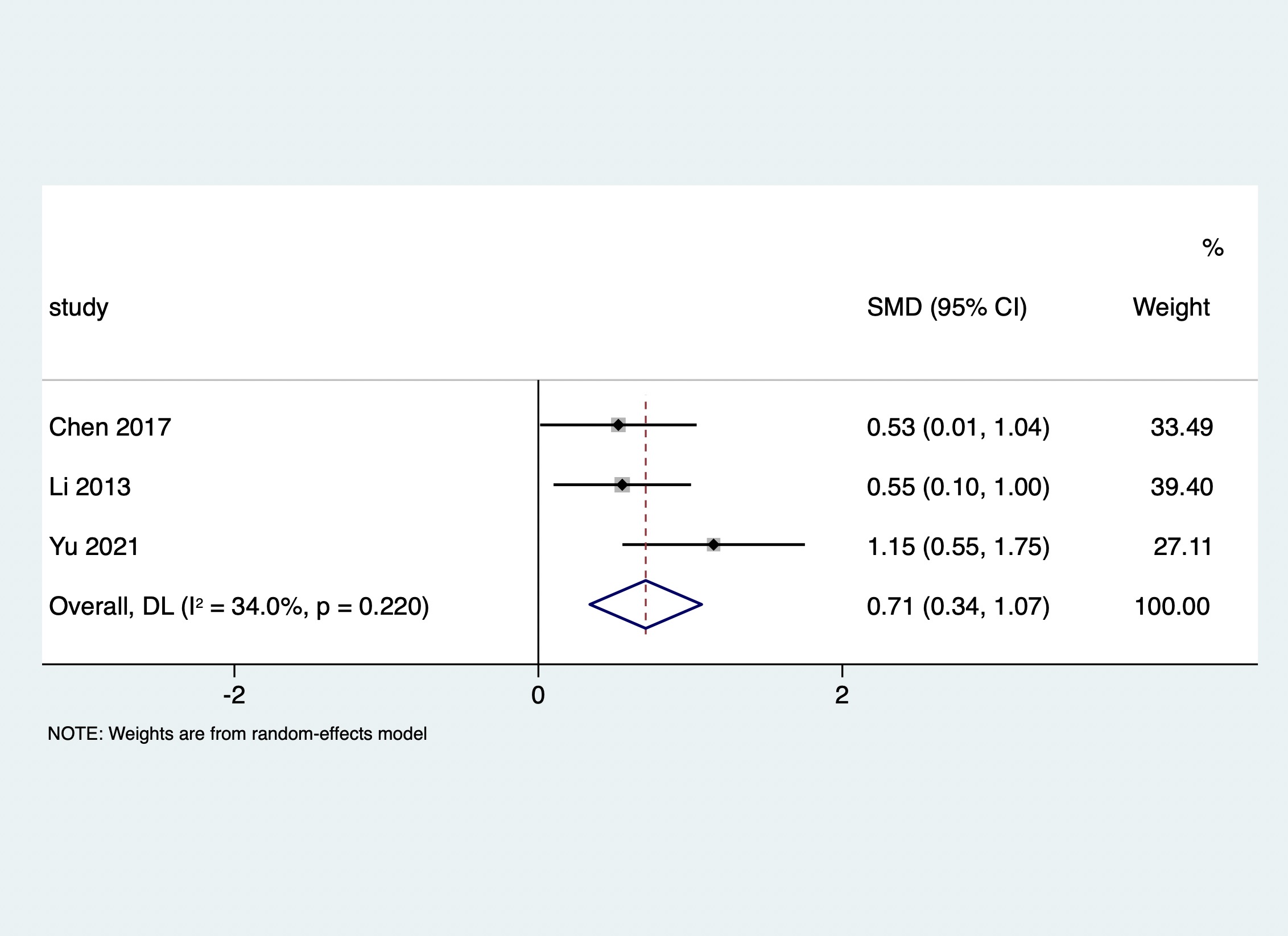

Supplement: Supplementary file 5 [file Image_5.jpeg]
