# Supplementary material for: Comparative efficacy of acupuncture-related techniques for mild cognitive impairment: A Bayesian network analysis
Source: Front Neurol. 2022 Nov 15;13:942682. doi: 10.3389/fneur.2022.942682 (PMC9706122; doi:10.3389/fneur.2022.942682)
Supplement: Supplementary file 6 [file Data_Sheet_1.docx]

Appendix 1: Search terms and search strategies for each database

**Pubmed Search strategy:**

**Search:**

#1 ("Cognitive Dysfunction"[Mesh])

#2 ((((((((((((((((((((((Cognitive Dysfunctions[Title/Abstract]) OR (Dysfunction, Cognitive[Title/Abstract])) OR (Dysfunctions, Cognitive[Title/Abstract])) OR (Cognitive Impairments[Title/Abstract])) OR (Cognitive Impairment[Title/Abstract])) OR (Impairment, Cognitive[Title/Abstract])) OR (Impairments, Cognitive[Title/Abstract])) OR (Mild Cognitive Impairment[Title/Abstract])) OR (Cognitive Impairment, Mild[Title/Abstract])) OR (Cognitive Impairments, Mild[Title/Abstract])) OR (Impairment, Mild Cognitive[Title/Abstract])) OR (Impairments, Mild Cognitive[Title/Abstract])) OR (Mild Cognitive Impairments[Title/Abstract])) OR (Mild Neurocognitive Disorder[Title/Abstract])) OR (Disorder, Mild Neurocognitive[Title/Abstract])) OR (Disorders, Mild Neurocognitive[Title/Abstract])) OR (Mild Neurocognitive Disorders[Title/Abstract])) OR (Neurocognitive Disorder, Mild[Title/Abstract])) OR (Neurocognitive Disorders, Mild[Title/Abstract])) OR (Cognitive Decline[Title/Abstract])) OR (Cognitive Declines[Title/Abstract])) OR (Mental Deteriorations[Title/Abstract]))

#3 #1 OR #2

#4 ((((randomized controlled trial[Title/Abstract]) OR (controlled trial[Title/Abstract])) OR (clinical trial[Title/Abstract])) OR (case-control studies[Title/Abstract])) OR (case series[Title/Abstract])

#5 ((((((((((((acupuncture[Title/Abstract]) OR (acupoints[Title/Abstract])) OR (body acupuncture[Title/Abstract])) OR (scalp acupuncture[Title/Abstract])) OR (manual acupuncture[Title/Abstract])) OR (Acupoint injection[Title/Abstract])) OR (autologous whole-blood acupoint injection[Title/Abstract])) OR (electroacupuncture[Title/Abstract])) OR (Autohemotherapy[Title/Abstract])) OR (fire needle[Title/Abstract])) OR (plum blossom needle[Title/Abstract])) OR (catgut implantation[Title/Abstract])) OR (filiform steel needle[Title/Abstract])

#6 #3 AND #4 AND #5

**EMBASE Search strategy**

#1 "Cognitive Dysfunction":ab,ti OR "Cognitive Dysfunctions":ab,ti OR "Dysfunctions, Cognitive":ab,ti OR "Dysfunction, Cognitive":ab,ti OR "Cognitive Impairments":ab,ti OR "Cognitive Impairment":ab,ti OR "Impairment, Cognitive":ab,ti OR "Impairments, Cognitive":ab,ti OR "Mild Cognitive Impairment":ab,ti OR "Cognitive Impairment, Mild":ab,ti OR "Cognitive Impairments, Mild":ab,ti OR "Impairment, Mild Cognitive":ab,ti OR "Impairments, Mild Cognitive":ab,ti OR "Mild Cognitive Impairments":ab,ti OR "Mild Neurocognitive Disorder":ab,ti OR "Disorder, Mild Neurocognitive":ab,ti OR "Disorders, Mild Neurocognitive":ab,ti OR "Mild Neurocognitive Disorders":ab,ti OR "Neurocognitive Disorder, Mild":ab,ti OR "Neurocognitive Disorders, Mild":ab,ti OR "Cognitive Decline":ab,ti OR "Cognitive Declines":ab,ti OR "Decline, Cognitive":ab,ti OR "Declines, Cognitive":ab,ti OR "Mental Deterioration":ab,ti OR "Deterioration, Mental":ab,ti OR "Deteriorations, Mental":ab,ti OR "Mental Deteriorations":ab,ti

#2 exp Mild cognitive impairment

#3 "randomized controlled trial":ab,ti OR "controlled trial":ab,ti OR "clinical trial":ab,ti OR "case-control studies":ab,ti OR "case series":ab,ti

#4 exp randomized controlled trial

#5 #1 OR #2

#6 #3 OR #4

#7 "filiform steel needle":ab,ti OR "body acupuncture":ab,ti OR "autologous whole-blood acupoint injection":ab,ti OR "scalp acupuncture":ab,ti OR "catgut implantation":ab,ti OR "electroacupuncture":ab,ti OR "manual acupuncture":ab,ti OR "acupoints":ab,ti OR "acupuncture":ab,ti OR "Acupoint injection":ab,ti

#8 exp Acupuncture

#9 #7 OR #8

#10 #5 AND #6 AND #9

**Web of Science Search strategy**

#1 TS=(Cognitive Dysfunction or Cognitive Dysfunctions or Dysfunction, Cognitive or Dysfunctions, Cognitive or Cognitive Impairments or Cognitive Impairment or Impairment, Cognitive or Impairments, Cognitive or Mild Cognitive Impairment or Cognitive Impairment, Mild or Cognitive Impairments, Mild or Impairment, Mild Cognitive or Impairments, Mild Cognitive or Mild Cognitive Impairments or Mild Neurocognitive Disorder or Disorder, Mild Neurocognitive or Disorders, Mild Neurocognitive or Mild Neurocognitive Disorders or Neurocognitive Disorder, Mild or Neurocognitive Disorders, Mild or Cognitive Decline or Cognitive Declines or Decline, Cognitive or Declines, Cognitive or Mental Deterioration or Deterioration, Mental or Deteriorations, Mental or Mental Deteriorations)

#2 TS=((randomized controlled trial) OR (controlled trial) OR (clinical trial) OR (case-control studies) OR (case series) )

#3 TS=((acupuncture∗) OR (acupuncture∗ Treatment∗) OR (acupuncture∗ therapy∗)

OR (body acupuncture∗) OR (Needle∗ acupuncture∗) OR (Manual∗ acupuncture∗) OR (acupuncture∗ Point∗) OR (electroacupuncture∗) OR (Warm∗ acupuncture∗) OR (electr∗-acupuncture∗) OR (acupoints) OR (Acupoint injection) OR (plum blossom needle) OR (filiform steel needle) OR (catgut implantation) OR (Autohemotherapy) OR (fire needle) OR (scalp acupuncture) OR (manual acupuncture) OR (autologous whole-blood acupoint injection) OR (electroacupuncture) OR (body acupuncture))

#4 #3 AND #2 AND #1

**Cochrane Library Search strategy**

#1 MeSH descriptor: [ Cognitive impairment] explode all trees

#2 (Cognitive Dysfunctions):ti,ab,kw OR(Dysfunction,Cognitive):ti,ab,kw

(Dysfunctions, Cognitive):ti,ab,kw OR (Cognitive Impairments):ti,ab,kw OR (Cognitive Impairment):ti,ab,kw OR (Impairment, Cognitive):ti,ab,kw

#3 (Impairments, Cognitive):ti,ab,kw OR (Mild Cognitive Impairment):ti,ab,kw OR (Cognitive Impairment, Mild):ti,ab,kw OR (Cognitive Impairments, Mild):ti,ab,kw OR (Impairment, Mild Cognitive):ti,ab,kw OR (Impairments, Mild Cognitive):ti,ab,kw OR (Mild Cognitive Impairments):ti,ab,kw OR (Mild Neurocognitive Disorder):ti,ab,kw OR (Disorder, Mild Neurocognitive):ti,ab,kw OR (Disorders, Mild Neurocognitive):ti,ab,kw OR (Mild Neurocognitive Disorders):ti,ab,kw OR (Neurocognitive Disorder, Mild):ti,ab,kw OR (Cognitive Decline):ti,ab,kw OR (Cognitive Declines):ti,ab,kw OR (Decline, Cognitive):ti,ab,kw OR (Declines, Cognitive):ti,ab,kw OR (Mental Deterioration):ti,ab,kw OR (Deterioration, Mental):ti,ab,kw OR (Mental Deteriorations):ti,ab,kw OR (Deteriorations, Mental):ti,ab,kw OR (Neurocognitive Disorders, Mild):ti,ab,kw

#4 #1 OR #2 OR #3

#5 MeSH descriptor: [Acupuncture] explode all trees

#6 (filiform steel needle):ti,ab,kw OR (body acupuncture):ti,ab,kw OR (autologous whole-blood acupoint injection):ti,ab,kw OR (scalp acupuncture):ti,ab,kw OR (catgut implantation):ti,ab,kw OR (electroacupuncture):ti,ab,kw OR (acupuncture):ti,ab,kw OR (manual acupuncture):ti,ab,kw OR (acupoints):ti,ab,kw OR (Acupoint injection):ti,ab,kw

#7 #5 OR #6

#8 MeSH descriptor: [randomized controlled trial] explode all trees

#9 (randomized controlled trial):ti,ab,kw OR (controlled trial):ti,ab,kw OR (clinical trial):ti,ab,kw OR (case-control studies):ti,ab,kw OR (case series):ti,ab,kw

#10 #8 OR #9

#11 #4 AND #7 AND #10

CNKI：

((SU='认知障碍' OR SU='轻度认知障碍' OR SU='轻型认知障碍' OR SU='认知损害' OR SU='早期认知障碍' OR SU='认知功能障碍' OR SU='老年型认知障碍' OR SU='老年轻度认知障碍 ' OR SU='老年型轻度认知障碍' OR SU='早期认知损害' OR SU='认知功能损害' OR SU='老年人早期认知障碍'))AND (SU='针刺'OR SU='针灸' OR SU='灸' OR SU='针法' OR SU='刺法' OR SU='体针' OR SU='腹针' OR SU='头针' OR SU='温针' OR SU='火针' OR SU='电针' OR SU='梅花针' OR SU='刺络' OR SU='放血' OR SU='拔罐' OR SU='耳穴' OR SU='穴位注射' OR SU='穴位按压' OR SU='穴位埋线') )AND (SU='试验' OR SU='随机' OR SU='对照'OR SU=‘临床研究’OR SU=‘临床观察’)

VIP：

((M=(认知障碍 + 轻度认知障碍 + 轻型认知障碍 + 认知损害 +早期认知障碍 + 认知功能障碍 + 老年型认知障碍 + 老年轻度认知障碍 + 老年型轻度认知障碍 + 早期认知损害 + 认知功能损害 + 老年人早期认知障碍 + 老年人轻度认知障碍) AND M =(针灸 + 针刺 + 灸 + 针法 + 刺法 + 体针 + 腹针 + 头针 + 温针 + 火针 + 电针 + 电磁针 + 梅花针 + 刺络 + 放血 + 拔罐 + 耳穴 + 穴位注射 + 穴位按压 + 穴位埋线)) AND R =(试验 + 随机 + 对照 + 临床研究 + 临床观察)

Wangfang：

(题名或关键词：(认知障碍 + 轻度认知障碍 + 轻型认知障碍 + 认知损害 +早期认知障碍 + 认知功能障碍 + 老年型认知障碍 + 老年轻度认知障碍 + 老年型轻度认知障碍 + 早期认知损害 + 认知功能损害 + 老年人早期认知障碍 + 老年人轻度认知障碍) )* 题名或关键词: (针灸 + 针刺 + 灸 + 针法 + 刺法 + 体针 + 腹针 + 头针 + 温针 + 火针 + 电针 + 电磁针 + 梅花针 + 刺络 + 放血 + 拔罐 + 耳穴 + 穴位注射 + 穴位按压 + 穴位埋线) * 摘要: (试验 + 随机 + 对照 + 临床研究 +临床观察)
